# Investigating the impact of type I-E CRISPR-Cas systems and *acrEI10* on multidrug-resistance in clinical isolates of *Klebsiella pneumoniae*

**DOI:** 10.1371/journal.pone.0335756

**Published:** 2025-11-19

**Authors:** Maryam Siroosi, Fatemeh Ghasemi, Fereshteh Jabalameli, Mohammad Emaneini, Mohammadreza Salehi, Reza Beigverdi, Mohammad Ali Amoozegar

**Affiliations:** 1 Department of Microbiology, School of Medicine, Tehran University of Medical Sciences, Tehran, Iran; 2 Extremophiles Laboratory, Department of Microbiology, School of Biology, College of Science, University of Tehran, Tehran, Iran; 3 Research Center for Antibiotic Stewardship and Antimicrobial Resistance, Tehran University of Medical Sciences, Tehran, Iran; 4 Research Center for Antibiotics Stewardship and Antimicrobial Resistance, Infectious Diseases Department, Imam Khomeini Hospital Complex, Tehran University of Medical Sciences, Tehran, Iran; Ahvaz Jundishapur University of Medical Sciences: Ahvaz Jondishapour University of Medical Sciences, IRAN, ISLAMIC REPUBLIC OF

## Abstract

*Klebsiella pneumoniae* is a pathogen related to nosocomial infections with a high rate of antibiotic resistance. The aim of this study was to understand the impact of the presence of CRISPR-Cas systems and an anti-CRISPR gene on multidrug-resistance in *K. pneumoniae* isolates. The study analyzed 100 clinical *K. pneumoniae* isolates obtained from a hospital setting. The investigation involved determining antibiotic resistance profiles, including ESBL production, identifying specific carbapenemase and aminoglycoside resistance genes, detecting the presence of CRISPR-Cas systems, identifying the anti-CRISPR gene *acrEI10*, and sequencing CRISPR arrays. Correlation analysis between resistance genes and CRISPR-Cas systems was also performed. All isolates in this study were determined to be multidrug-resistant (MDR), with resistance rates exceeding 70% for the majority of antibiotics tested. The most prevalent carbapenemase genes were *bla*_OXA-48_ and *bla*_NDM_, while aminoglycoside resistance was primarily mediated by *aac(*6*´)-Ia* and *ant(2")-Ia*. Only 7% of the isolates harbored CRISPR-Cas systems and the gene *acrEI10*, which encodes an anti-CRISPR protein, was detected in one of the CRISPR-Cas positive isolates. Sequencing of the CRISPR array from this isolate showed similarities between the spacers and sequences found in plasmids and *K. pneumoniae* chromosome. No strong correlation was identified between the antibiotic resistance genes and CRISPR-Cas systems. Findings from this study suggest a complex interplay between these factors in MDR isolates of *K. pneumoniae* and show that further investigations are needed to have a better understanding of the mechanisms related to the coexistence of these elements and their impact on dissemination of antibiotic resistance genes.

## Introduction

*Klebsiella* *pneumoniae*, a member of *Enterobacteriaceae* family, is a ubiquitous Gram-negative bacterium and a threat to hospital settings. This pathogen is known to cause various nosocomial infections, including pneumonia, urinary tract infections, and bloodstream infections [1]. Studies have shown that factors such as prior exposure to certain antibiotics, underlying medical conditions (e.g., immunodeficiency), and admission to intensive care units (ICUs) contribute to increased risk of *K. pneumoniae* colonization and infection. Management of these infections is further complicated by *K. pneumoniae’s* increasing resistance to commonly used antibiotics, including third generation cephalosporins, aminoglycosides, and even carbapenems, which are often considered last-resort treatments [2]. Indeed, global prevalence studies highlight the severity of this issue. For instance, resistance to aminoglycosides and *β*-lactams in multidrug-resistant *K. pneumoniae* isolates from nosocomial infections has been reported at alarming rates of 85.1% and 91.5%, respectively, underscoring its status as a worldwide concern [3]. These alarming rates of resistance are primarily attributed to several key mechanisms, including reduced permeability to antibiotics, overexpression of efflux pumps, and the production of antibiotic hydrolyzing/modifying enzymes within this bacterium [4]. Notably, resistance to aminoglycosides and carbapenems is often mediated by plasmid-encoded enzymes, facilitating their transfer between bacteria [2]. The acquisition and dissemination of these plasmids carrying resistance genes can, however, be influenced by bacterial defense mechanisms such as the CRISPR-Cas system. Clustered regularly interspaced short palindromic repeats (CRISPR-Cas systems) are one of the prokaryotic defense mechanisms against invading genetic elements like plasmids. CRISPR-Cas loci contain a set of CRISPR-associated (*cas*) genes which encode Cas nucleases involved in cleaving foreign nucleic acids and arrays of short, repetitive sequences interspersed by variable sequences known as spacers [5]. These spacers are sequences acquired from mobile genetic elements (MGEs), such as phages, transposons, and plasmids. CRISPR-Cas as an immune system is able to recognize and cleave invasive nucleic acids which the host has encountered before and stored part of their sequence as spacers [6]. However, the discovery of anti-CRISPR (Acr) proteins within MGEs has introduced a new layer of complexity to this defense mechanism. Acr proteins specifically inhibit Cas nucleases, enabling MGEs to evade CRISPR-Cas defense and promote their own dissemination; they are also responsible for bacterial survival against self-targeting CRISPR-Cas systems [7]. A newly identified, broad-spectrum Acr protein obtained from an MDR isolate, AcrEI10, has shown inhibitory effect against CRISPR-Cas system type IE in *K. pneumoniae* and proposed a potential role in spreading resistance between bacteria [8].

Recent studies have explored the possible relationship between antibiotic resistance genes and CRISPR-Cas systems in bacteria. Some investigations have suggested an inverse correlation between these two elements, proposing that CRISPR-Cas systems may inhibit the acquisition of antibiotic resistance genes carried on MGEs [9–12]. However, other studies have found no significant association [13–15]. The discovery of Acr proteins, which can inhibit CRISPR-Cas activity, allows MGEs to evade CRISPR-Cas defense mechanisms and facilitate the spread of antibiotic resistance genes, further complicates the effectiveness of this immune system in preventing the antibiotic resistance in bacteria [7]. Despite growing interest, there remains a critical gap in understanding the precise interplay between clinically relevant antibiotic resistance genes (carbapenemase and aminoglycoside-modifying enzyme genes which inactivate two important classes of antibiotics), CRISPR-Cas systems (especially type I-E and I-E* as typical CRISPR-Cas systems in *K. pneumoniae* [16]), and Acr proteins (especially the broad-spectrum AcrEI10) within multidrug resistant *K. pneumoniae* clinical isolates.

This study investigates the presence of a number of more prevalent carbapenemase genes (*bla*_VIM_, *bla*_NDM_, *bla*_KPC_, and *bla*_OXA-48_), aminoglycoside-modifying enzyme genes (*aph(3´)-Ia*, *aac(6´)-Ia*, *ant(2")-Ia*, *aac(3)-IIa*, *ant(4´)-IIa*, *aac(3)-IVa*, and *aac(3)-Ia*), as well as the most common types of CRISPR-Cas system in *K. pneumoniae* (I-E CRISPR1, I-E* CRISPR2, and I-E* CRISPR3) along with *cas1* and *cas3* genes in 100 multidrug-resistant *K. pneumoniae* isolates from a teaching hospital in Tehran, Iran. Also, the primers for detecting a newly discovered Acr gene in *K. pneumoniae*, *acrIE10*, were designed and used to detect this gene. Additionally, the CRISPR array sequence of one isolate harboring a CRISPR-Cas system, *acrIE10*, and antibiotic resistance genes was analyzed.

## Materials and methods

### Bacterial isolates

A total of 100 clinically identified *K. pneumoniae* isolates were collected via a convenience sampling strategy from the clinical microbiology laboratory of Imam Khomeini Hospital Complex (a teaching and referral hospital of Tehran University of Medical Sciences) between September 2023 and February 2024. This meant that consecutively available isolates meeting the study’s criteria were received during this six-month period. Inclusion criteria for obtaining the isolates were defined as follows: isolates from inpatients of both sexes and any age, suffering from hospital-acquired infections. These isolates could originate from any relevant clinical sample type, including urine, tracheal aspirates, blood, body fluids, wound swabs, and other clinical specimens. Isolates from any hospital ward (including ICUs, surgical, general, oncology, neurology, and other clinical wards) were considered. To ensure independence of samples, only one isolate per patient was included. Exclusion criteria comprised isolates from outpatients or community-acquired infections, duplicate isolates from the same patient, and isolates with incomplete biochemical identification.

Molecular confirmation of the isolates was performed by targeting the *khe* gene, a specific marker for *K. pneumoniae*, using the PCR method [17]. Genomic DNA was extracted from bacterial colonies grown on LB agar by the boiling method and used as the template in a 25 μl PCR reaction mixture containing 12.5 μl of 2x master mix red (Ampliqon, Denmark), 2.5 μl of each forward and reverse primer (final concentration: 1 μM), 2 μl extracted DNA, and 5.5 μl distilled water. Amplification was performed using the following cycling conditions: initial denaturation at 94°C for 4 min, followed by 30 cycles of denaturation at 94°C for 1 minute, annealing at 58°C for 30 s, extension at 72°C for 30 s, and a final extension at 72°C for 7 min.

### Antimicrobial susceptibility testing

Antimicrobial susceptibility of the isolates towards antibiotics was determined by the disk diffusion method, following the Clinical and Laboratory Standards Institute (CLSI) guidelines [18] and the isolates were stored at −70ºC. According to Magiorakos *et al*., if an isolate is not susceptible to at least one agent in three or more antibiotic categories, the isolate is considered as multidrug-resistant or MDR [19]. Zone diameter breakpoints (mm) for interpretation were based on CLSI guidelines and were as follows: ampicillin (sensitive ≥ 17, intermediate 14–16, resistant ≤ 13), co-trimoxazole (sensitive ≥ 16, intermediate 11–15, resistant ≤ 10), ciprofloxacin (sensitive ≥ 26, intermediate 22–25, resistant ≤ 21), ceftazidime (sensitive ≥ 21, intermediate 18–20, resistant ≤ 17), gentamicin (sensitive ≥ 15, intermediate 13–14, resistant ≤ 12), and imipenem (sensitive ≥ 23, intermediate 20–22, resistant ≤ 19).

### Phenotypic detection of extended-spectrum *β*-lactamases (ESBLs)

Isolates were further examined for ESBL production using the double-disk synergy phenotypic confirmatory method, in accordance with the CLSI guidelines. For this purpose, ceftazidime and ceftazidime/clavulanic acid disks were employed. Isolates were classified as ESBL producers if the inhibition zone diameter around the ceftazidime/clavulanic acid disk was ≥ 5 mm larger than that around the ceftazidime disk alone.

### Molecular detection of antibiotic resistance genes

The PCR method was used to detect carbapenemase genes including *bla*_VIM_, *bla*_NDM_, *bla*_KPC_, and *bla*_OXA-48_ and aminoglycoside-modifying enzyme genes including *aph(*3*´)-Ia*, *aac(*6*´)-Ia*, *ant(*2*")-Ia*, *aac(*3)-*IIa*, *ant(*4*´)-IIa*, *aac(*3)-*IVa*, and *aac(*3)-*Ia* [20]. Each target gene was amplified in a separate monoplex PCR reaction. Genomic DNA, extracted by the boiling method, was used as the template in a 25 μl PCR reaction mixture prepared as outlined in bacterial isolates section. Specific primer sets were used for each target gene. Each PCR assay was optimized by determination of optimal annealing temperatures. Validation included the consistent use of positive and negative controls for each gene, and confirmation of correct amplicon size via agarose gel electrophoresis. Annealing temperatures, extension times, and amplicon sizes varied for each primer set and are also detailed in S1 Table.

### CRISPR-Cas detection and DNA sequencing

The presence of CRISPR arrays including I-E CRISPR1, I-E* CRISPR2, and I-E* CRISPR3 and *cas1* and *cas*3 genes was determined by PCR using the general method mentioned in the bacterial isolates section with specific annealing temperatures and extension times for each primer set detailed in S1 Table. The primer sequences are listed in Table 1. Purified PCR product of I-E^*^ CRISPR3 array amplification from the isolate Kp166 was sent to Microsynth Seqlab (Goettingen, Germany) for Sanger sequencing.

**Table 1:** Primer sequences and amplicon sizes for CRISPR arrays and *cas1*, *3*, and *acrIE10* genes.

Target	Primer sequence (5´-3´)	Amplicon size (bp)	Reference
*cas1*	F-GCTGTTTGTCAAAGTTACCCGCGAACTC R-GGTTTTGATCGCCTCATGAGTCACAGTTG	208	[21]
*cas3*	F-TGGCCGACATTTGATTCAGC R-CCATGCTTAACATTCATCAC	620	[22]
I-E CRISPR1	F-CAGTTCCTGCAACCTGGCCT R-CTGGCAGCAGGTGATACAGC	Variable	[21]
I-E^*^ CRISPR2	F-GTAGCGAAACCCTGATCAAGCG R-GCGCTACGTTCTGGGGATG	Variable	[21]
I-E^*^ CRISPR3	F-GACGCTGGTGCGATTCTTGAG R-CGCAGTATTCCTCAACCGCCT	Variable	[21]
*acrIE10*	F-AGCTGCGAGCACATCTGAAC R-CAGATAATCATCCGAGTCCCG	212	This study

### Anti-CRISPR detection

In order to design primers to detect *acrIE10*, a novel anti-CRISPR gene in drug-resistance plasmids of *K. pneumoniae*, a BLASTN search was performed against the NCBI nucleotide database using AcrIE10 encoding gene (Gene bank accession number: OQ389520.1) as the query. The top 100 hits were aligned using Clustal Omega [23] and conserved regions were identified by visual inspection. According to the conserved regions of the gene, forward and reverse primers were designed and PCR method was used to detect this gene in all CRISPR-Cas positive isolates using the general method mentioned in the bacterial isolates section with specific annealing temperature and extension time for each primer set detailed in S1 Table. The sequences of the primers are shown in Table 1, and their specificity and absence of off-target binding were confirmed using BLASTN and Primer–BLAST [24].

### CRISPR array sequence analysis

CRISPRCasFinder webserver with the default settings [25] was used to analyze I-E^*^ CRISPR3 array sequenced from the isolate Kp166. All CRISPR spacers found by the server were subjected to NCBI Microbial Nucleotide BLAST search (with default parameters) to determine the origin of the spacers.

### Statistical analysis

Correlation between the presence of antibiotic resistance genes and CRISPR-Cas system was determined by Spearman’s rank correlation coefficient test (GraphPad Prism) with an alpha set at 0.05.

## Results

### Source of isolates and antibiotic resistance profile

In this study, 100 clinically identified *K. pneumoniae* isolates were collected and all of them were positive for the *khe* gene. The age of patients from whom the specimens were collected ranged from one month to 94 years old, with an average of 51.8 ± 19.7 years. 49 isolates were collected from females and 51 isolates were collected from males. The distribution of the isolate sources is presented in Table 2.

**Table 2:** Distribution of isolate sources for 100 *K. pneumoniae* isolates.

Type of specimen	Number (n)
Urine	45
Trachea	16
Body fluids	13
Blood	11
Wound	6
Other specimens	9
Total	100

A high prevalence of multidrug resistance was observed, with 66% (n = 66) of the isolates resistant to all tested antibiotics and 90% (n = 90) resistant to at least three different antibiotic categories, classifying them as MDR. Urine specimens accounted for the highest number of MDR isolates (42%, n = 42), followed by trachea (17%, n = 17), body fluids (13%, n = 13), blood (11%, n = 11), other specimens (10%, n = 10), and wound specimens (7%, n = 7). However, when considering the proportion of MDR isolates within each specimen type, wound and other specimens had the highest rates of MDR, with 100% of isolates (n = 6 and 9, respectively) in these categories exhibiting multidrug resistance. Trachea, body fluids, and blood specimens also had relatively high rates of MDR, with 94% (n = 15), 92% (n = 12), and 90% (n = 10) of isolates, respectively. Urine specimens, while accounting for the largest number of MDR isolates overall, had a slightly lower MDR prevalence of 84% (n = 38).

**Fig 1:**
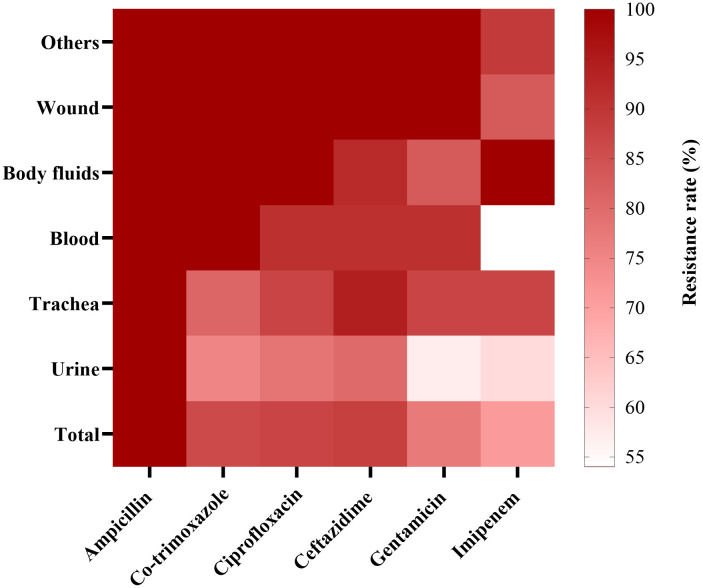
Heatmap representation of antibiotic resistance rates of *K. pneumoniae* isolates (n = 100) and types of specimens. The heatmap represents the percentage of isolates resistant to a panel of six antibiotics: Ampicillin, Co-trimoxazole, Ciprofloxacin, Ceftazidime, Gentamicin, and Imipenem. Rows represent different specimen types (Urine, Trachea, Blood, Body fluids, Wound, Others) and columns represent individual antibiotics. The color scale reflects the proportion of resistant isolates for each antibiotic and specimen type combination, ranging from white (low resistance) to dark red (high resistance).

Antibiotic resistance profiles of the isolates from each specimen are visualized in Fig 1. According to this heatmap, isolates recovered from other specimens and wound specimens showed the highest resistance rates to all tested antibiotics compared to other types of specimens. In contrast, urine isolates exhibited the lowest overall antibiotic resistance.

Resistance to ampicillin, ceftazidime, co-trimoxazole, ciprofloxacin, gentamicin, and imipenem was observed in 98, 89, 86, 91, 78, and 73% of isolates, respectively. Totally, 52% of all isolates (52 isolates out of total 100 isolates) and 64% of ceftazidime-resistant isolates (52 out of 89 ceftazidime-resistant isolates) were ESBL producers. Demographic and source data for each isolate, including patient age and sex, and specimen type and hospital ward are presented in the S1 Data.

### Distribution of antibiotic resistance genes in isolates

Tables 3 and 4 summarize the prevalence of carbapenemase and aminoglycoside-modifying enzyme genes, respectively, among 100 studied clinical isolates. These tables also detail the corresponding antibiotic resistance rates among gene-positive isolates. The *ant(*2*")-Ia* gene was the most prevalent among all tested genes, detected in 65% of isolates, followed by the *bla*_OXA-48_ gene (55%).

**Table 3:** Prevalence of carbapenemase genes and associated resistance to imipenem in the 100 *K. pneumoniae* isolates.

Carbapenemase gene	Prevalence (n^*^,)	Imipenem resistance (n^†^, %)
*bla*_VIM_	0, 0	N/A^**‡**^
*bla*_NDM_	32, 32	29, 91
*bla*_KPC_	0, 0	N/A
*bla*_OXA-48_	55, 55	51, 93
*bla*_NDM _+ *bla*_OXA-48_	26, 26	24, 92

* Number of isolates positive for antibiotic resistance genes in the total of 100 isolates tested.

† Number of imipenem resistant isolates in the isolates positive for carbapenemase genes.

‡ Not applicable.

Among the 73 isolates phenotypically resistant to imipenem, 84% were positive for at least one carbapenemase gene. Similarly, 88% of the 78 isolates resistant to gentamicin were tested positive for at least one aminoglycoside-modifying enzyme gene. Notably, isolates co-carrying *bla*_OXA-48_ and *bla*_NDM_ demonstrated 100% resistance to ceftazidime.

**Table 4:** Prevalence of aminoglycoside-modifying enzymes and associated resistance to gentamicin in the 100 *K. pneumoniae* isolates.

Aminoglycoside-modifying enzyme gene	Prevalence (n^*^, %)	Gentamicin resistance (n^†^, %)
*aph(3´)-Ia*	6, 6	2, 33
*aac(6´)-Ia*	48, 48	46, 96
*ant(2″)-Ia*	65, 65	55, 85
*aac(3)-IIa*	8, 8	8, 100
*ant(4´)-IIa*	0, 0	N/A^**‡**^
*aac(3)-IVa*	0, 0	N/A
*aac(3)-Ia*	0, 0	N/A
*aph(3´)-Ia *+ *aac(6´)-Ia*	1, 1	1, 100
*aph(3´)-Ia *+ *ant(2″)-Ia*	4, 4	2, 50
*aph(3´)-Ia *+ *aac(3)-IIa*	0, 0	N/A
*aac(6´)-Ia *+ *ant(2″)-Ia*	21, 21	20, 95
*aac(6´)-Ia* + *aac(3)-IIa*	7, 7	7, 100
*ant(2″)-Ia *+ *aac(3)-IIa*	3, 3	3, 100
*aph(3´)-Ia *+ *aac(6´)-Ia *+ *ant(2″)-Ia*	1, 1	1, 100
*aph(3´)-Ia *+ *aac(6´)-Ia *+ *aac(3)-IIa*	0, 0	N/A
*aac(6´)-Ia *+ *ant(2″)-Ia *+ *aac(3)-IIa*	3, 3	3, 100
*aph(3’)-Ia *+* ant(2″)-Ia* + *and aac(3)-IIa*	0, 0	N/A
*aph(3´)-Ia *+ *aac(6´)-Ia *+ *ant(2″)-Ia *+ *aac(3)-IIa*	0, 0	N/A
Any aminoglycoside-modifying enzyme gene	82, 82	69, 84

* Number of isolates positive for antibiotic resistance genes in the total of 100 isolates tested.

† Number of gentamicin resistant isolates in isolates positive for aminoglycoside-modifying enzyme gene.

‡ Not applicable.

**Fig 2:**
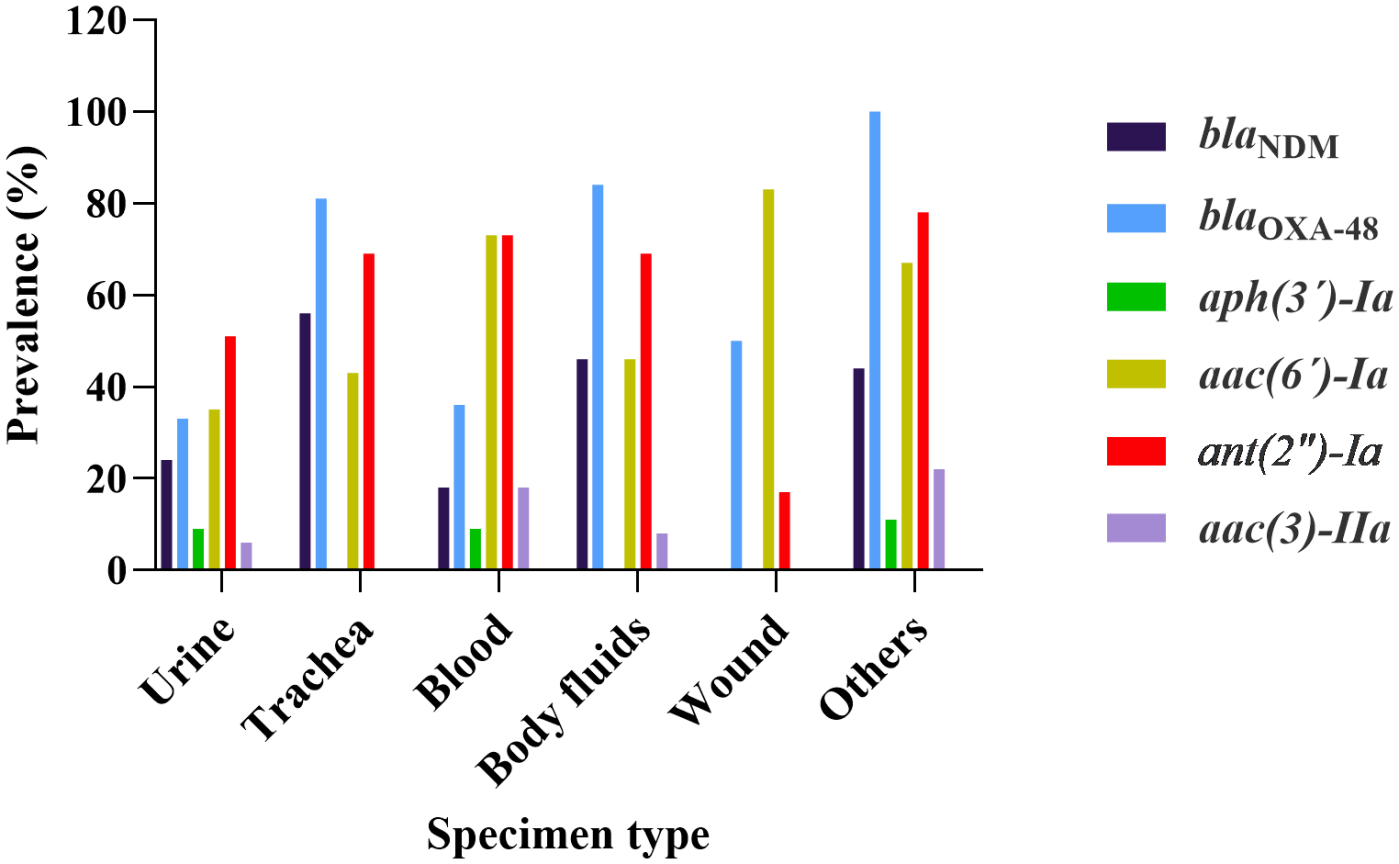
Specimen-dependent distribution of antibiotic resistance genes in *K. pneumoniae* isolates. The bar chart shows the prevalence (%) of specific resistance genes in isolates, categorized by specimen type (X-axis: Urine, Trachea, Blood, Body fluids, Wound, Others). The genes presented include carbapenem resistance genes (*bla*_NDM_, *bla*_OXA-48_) and aminoglycoside resistance genes (*aph(*3*’)-Ia*, *aac(*6*’)-Ia*, *aac(*3)-*IIa*, *ant(2″)-Ia*). The Y-axis represents the percentage of isolates within each specimen type that carry each indicated gene.

Fig 2 illustrates the distribution of antibiotic resistance genes across different specimen types. Genes *aac(*6*´)-Ia*, *bla*_OXA-48_, and *ant(2″)-Ia* were detected in all specimen types. Notably, *aac(*6*´)-Ia* and *bla*_OXA-48_ were present in at least 30% of isolates from each specimen type. Moreover, *bla*_NDM_ was found in all specimen types except for the wound.

### CRISPR-Cas systems in isolates

**Fig 3:**
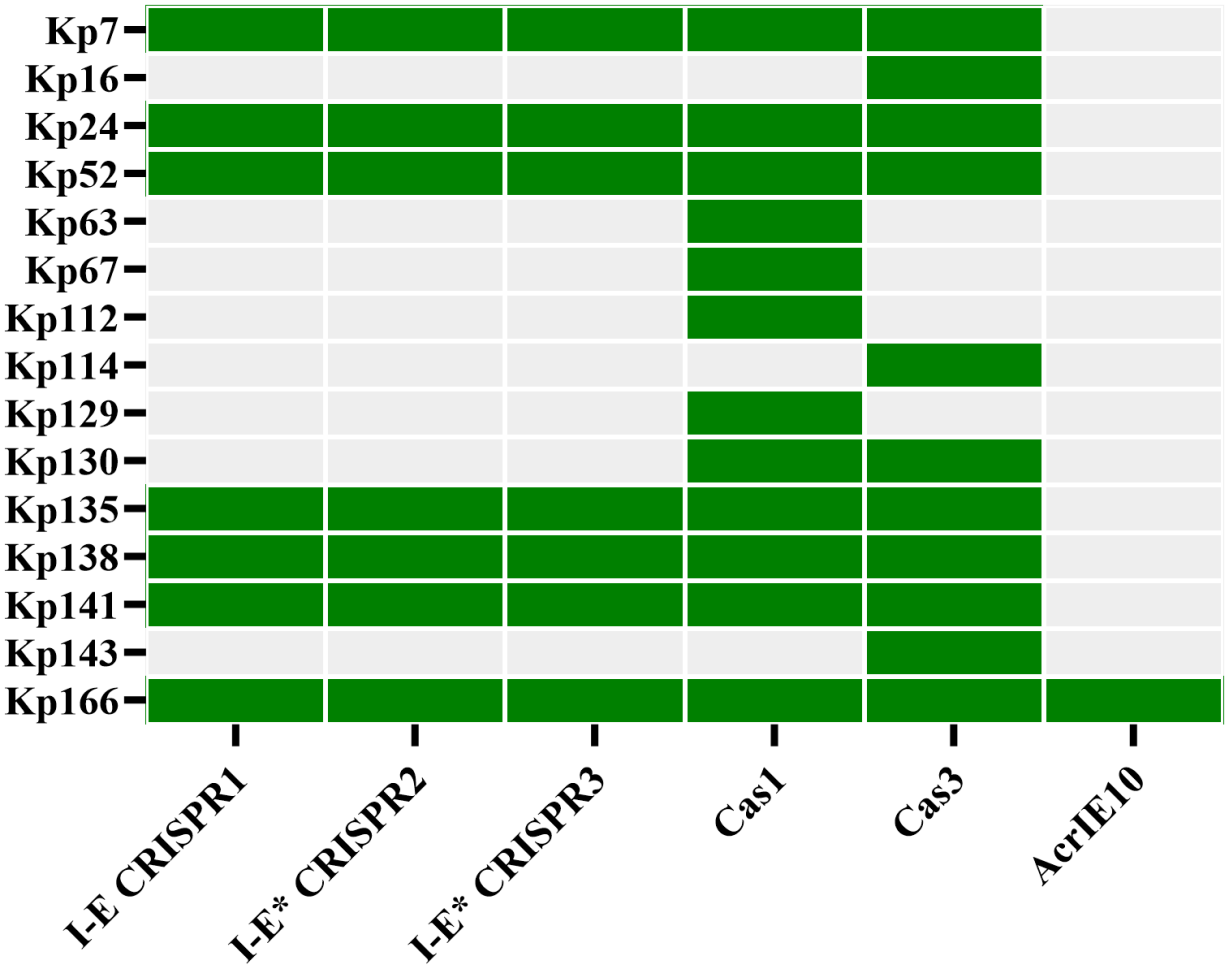
Distribution of CRISPR-Cas components and AcrIE10 encoding gene in *K. pneumoniae* isolates. The figure is a presence/absence matrix showing the distribution of CRISPR-Cas components in *K. pneumoniae* clinical isolates. Each row corresponds to an isolate (labeled Kp7-Kp166). Columns represent three putative Type I-E CRISPR arrays (I-E CRISPR1, I-E* CRISPR2, I-E* CRISPR3), two Cas genes (*cas1*, *cas3*), and AcrIE10 gene. Green indicates presence of the component; gray indicates absence.

Table 5 presents the prevalence of CRISPR arrays and Cas genes in 100 clinical isolates. Only 7% (n = 7) of the 100 isolates contained CRISPR arrays. All of these seven CRISPR-Cas system positive isolates simultaneously harbored I-E CRISPR1, I-E^*^ CRISPR2, and I-E^*^ CRISPR3, which were found in conjunction with *cas1* and *cas3* genes. As it is seen in Fig 3, among 100 isolates, four isolates possessed only the *cas1* gene, three had only the *cas3* gene, and one had both *cas1* and *cas3* genes. These isolates were classified as CRISPR-Cas system negative. Among the CRISPR-Cas system positive isolates (n = 7), only one harbored *acrIE10*.

**Table 5:** Prevalence of CRISPR arrays and Cas genes in the 100 *K. pneumoniae* isolates.

CRISPR-Cas System		Prevalence (%)
CRISPR array	I-E CRISPR1	7
	I-E^*^ CRISPR2	7
	I-E^*^ CRISPR3	7
Cas genes	*cas1*	12
	*cas3*	11

### CRISPR array spacer analysis

According to the results from CRISPRCasFinder webserver, the sequenced CRISPR array in the isolate Kp166 contained six direct repeats (each 28-nucleotide) with the consensus sequence of 5’-GAAACACCCCCACGTGCGTGGGGAAGAC-3’ and five unique spacers. BLASTN analysis indicated that one of the spacers shared homology with a known plasmid, while the remaining four showed homologies to the *K. pneumoniae* chromosome (Table 6). No homology was found to any known *K. pneumoniae* bacteriophages.

**Table 6:** The origin of spacers identified by BLASTN using default settings.

Spacer Sequence (5´-3´)	The Origin of Spacers	Identity (%)	E Value
TGCCGGATATCATCACCGCGATTAAACGGCGGA	Plasmid	100	6e-10
AGAACGAATGCCAGCGCTGGTACGGCGCGTCGTGGATTCCA	*K. pneumoniae* chromosome	100	6e-07
CATTCATAACAAAACCGCTTTTACTAGATGAGG	*K. pneumoniae* chromosome	100	6e-07
GCGGTGAACCTTGGGGCGTCTCCTCGCGAACTA	*K. pneumoniae* chromosome	100	6e-07
GCTACTGCATCCACGGCGTACATGCTCAGTGTA	*K. pneumoniae* chromosome	100	6e-07

### Correlation between antibiotic resistance genes and CRISPR-Cas system

**Fig 4:**
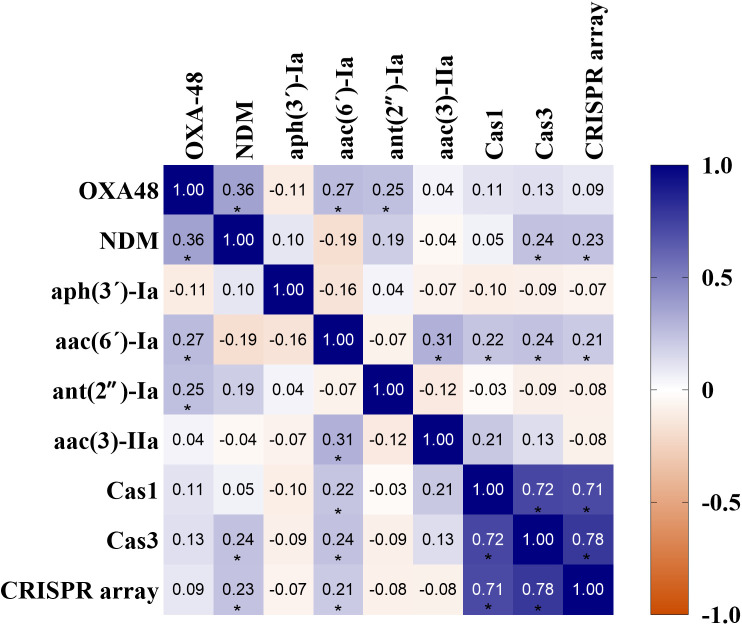
Correlation matrix of antibiotic resistance genes and CRISPR-Cas system components. Heatmap displays the correlation between the abundance of specific antibiotic resistance genes (*bla*_OXA-48_, *bla*_NDM_, *aph(*3*’)-Ia*, *aac(*6*’)-Ia*, *ant(2″)-Ia*, *aac(*3)-*IIa*), CRISPR-associated protein-encoding genes (Cas genes: Cas1, Cas3), and CRISPR arrays. Positive correlations (blue colors) indicate a tendency for these genes/features to co-occur, while negative correlations (orange colors) indicate inverse relationships. The intensity of the color corresponds to the strength of the correlation. Each cell contains the Pearson correlation coefficient (r-value), and statistically significant correlations are indicated with asterisks (*p* < 0.05).

Fig 4 illustrates the correlation between antibiotic resistance genes, Cas genes, and CRISPR arrays. As mentioned before, only isolates which contained Cas genes and CRISPR arrays were considered as CRISPR-Cas positive isolates (seven out of 100 isolates). According to this correlation matrix, no strong correlation was observed between antibiotic resistance genes and CRISPR-Cas system (Spearman’s rank correlation coefficients near to zero). However, a weak positive and significant correlation was detected between the CRISPR-Cas system and antibiotic resistance genes *bla*_NDM_ (r-value = 0.23; *p* < 0.05) and *aac(*6*´)-Ia* (r-value = 0.21; *p* < 0.05).

## Discussion

The relationship between bacterial CRISPR-Cas systems and the presence of antibiotic resistance genes in various pathogens is a subject of active and often conflicting research. While some studies report an inverse correlation, suggesting CRISPR-Cas systems actively limit resistance gene acquisition and dissemination [26], others have found a positive association or co-occurrence, indicating that CRISPR-Cas is not always a barrier, or may even be present in highly resistant strains [13]. This ongoing debate underscores the complexity of their interplay, influenced by factors such as Acr proteins, which interfere with the CRISPR-Cas system. Acr proteins facilitate the spread of antibiotic resistance genes by allowing MGEs to evade CRISPR-Cas defense. It shows more studies are needed to be conducted regarding antibiotic resistance genes, CRISPR-Cas system, and anti-CRISPR genes. To further understand the mechanisms of antibiotic resistance in *K. pneumoniae*, the prevalence of various resistance genes and CRISPR-Cas systems in a collection of clinical isolates was investigated.

In this study, 100 clinical isolates of *K. pneumoniae* from the clinical microbiology laboratory of Imam Khomeini Hospital Complex, a teaching and referral hospital in Tehran, Iran, were analyzed. Based on the antibiotic resistant profile of these isolates, a high prevalence of MDR was observed, with 90% of the isolates exhibiting resistance to multiple antibiotics. This finding aligns with recent studies on hospital-isolated clinical isolates of *K. pneumoniae* in different regions in Iran, which have reported similarly high MDR rates of 76% in Ahvaz (n = 106) and 85% in Azerbaijan (n = 100) [13,27]. Although the antibiotic exposure status of the patients was unknown in the present study, this high observed antibiotic resistance among the isolates could be attributed to factors inherent to the setting of a referral hospital which are characterized by a patient population with increased disease severity, necessitating the frequent use of broad-spectrum antibiotics. This elevated antibiotic selective pressure within the hospital environment promotes the emergence and persistence of resistant bacterial strains. Furthermore, in the current study, antibiotic resistance rates exceeded 70% for all tested antibiotics, including ampicillin, ceftazidime, co-trimoxazole, ciprofloxacin, gentamicin, and imipenem. The highest resistance rate was observed against ampicillin (98%), while the lowest was observed against imipenem (73%). These results are consistent with similar studies in Iran, which have also reported high levels of antibiotic resistance in *K. pneumoniae* isolates. For instance, Sharahi et al. (2021) found antibiotic resistance rates of over 65% for ceftazidime, co-trimoxazole, ciprofloxacin, gentamicin, and imipenem (n = 52; source of the isolates, hospital) [28], while Ghamari et al. (2022) observed resistance rates exceeding 75% for ceftazidime, ciprofloxacin, gentamicin, and imipenem (n = 60; source of the isolates, hospital) [20]. These similarities show the similar pattern in antibiotic use across Iranian hospitals and reflect a national trend. Also, 52% of all isolates in this study were ESBL producers according to the double-disk synergy, which was consistent with findings from other studies on clinical *K. pneumoniae* isolates in Middle Eastern countries [11,29].

According to a comprehensive review examining carbapenemase prevalence in the Middle East, *K. pneumoniae* is the most common carbapenemases-producing Enterobacterales across countries in this region, including Iran. Among the carbapenemases, OXA-48 is the most prevalent, followed by NDM, while KPC and VIM are less common [30]. In the present study, from studied carbapenemase genes, *bla*_OXA-48_ and *bla*_NDM_ were detected, with no detection of *bla*_VIM_ and *bla*_KPC_. This pattern aligns with prior studies in Iran, where *bla*_OXA-48_ and *bla*_NDM_ were the most frequently identified carbapenemase genes, and *bla*_VIM_ and *bla*_KPC_ were either absent or detected at very low rates [20,27,28,31]. Regarding aminoglycoside resistance in the isolates, genes *ant(2")-Ia* and *aac(*6*´)-Ia* were the most prevalent, with lower frequencies of *aph(*3*´)-Ia* and *aac(*3)-*IIa*, while *ant(*4*´)-IIa*, *aac(*3)-*IVa,* and *aac(*3)-*Ia* were not detected. This distribution is consistent with findings from other studies conducted in Iran, although prevalence rates for aminoglycoside resistance genes vary across different reports [20,27]. It should be noted that carbapenemases and aminoglycoside-modifying enzymes are not the sole mechanisms of resistance against carbapenems and aminoglycosides in *Enterobacteriaceae*. Other resistance mechanisms, such as efflux pumps or alterations in membrane permeability, can also contribute to resistance, depending on the antibiotic type [32]. This helps explain the observed discrepancies between phenotypic and genotypic antibiotic resistance profiles in this study, where the presence of antibiotic resistance genes did not necessarily translate to phenotypic antibiotic resistance.

CRISPR-Cas system is a type of immune system found in prokaryotes that protects them from invading genetic elements (e.g., plasmids and phages). These systems recognize and destroy foreign DNA by utilizing spacers to guide Cas proteins to matching sequences on the invading genetic elements. While these systems are effective against harmful invaders, like lytic phages, they can be less beneficial when the presence of these genetic elements is necessary for the survival of the cell, like when they contain antibiotic resistance genes and the cell is under antibiotic pressure. This creates a critical problem for bacteria, as the same system they use to protect themselves can also block them from getting helpful new abilities. In response to this problem, some bacteria lose this defense mechanism, while others might repress it [33]. That could explain why some studies have reported an inverse correlation between CRISPR-Cas system and antibiotic resistance genes in *K. pneumoniae* and some other bacteria [34], yet others have shown that these systems can coexist with antibiotic resistant genes. This co-presence is most probably because of the Acr proteins which inhibit CRISPR-Cas activity by blocking Cas proteins, although there might be some other factors allowing this coexistence [8], such as mutations in CRISPR-Cas system or CRISPR-Cas system repression when bacteria are under antibiotic pressure which helps them to acquire resistance genes, and the R-M (restriction-modification) systems which also controls the presence of plasmids in the cell [14,15]. It seems the activity of the CRISPR-Cas system is not conducive to bacterial survival in environments with high antibiotic pressure, which has led them to find solutions like Acr proteins and mutations in *cas* genes. All these anti-CRISPR-Cas system mechanisms help bacteria to acquire foreign DNA to withstand antibiotics and promote the dissemination of those genes to other bacteria [35]. Even the regulatory activity of the CRISPR-Cas system on bacterial physiology has been shown to be responsible for specific antibiotic resistance [36]. Moreover, some CRISPR arrays in bacteria contain self-targeting spacers and they need the presence of an MGE encoding an Acr protein to survive its own immune system [7]. A recent study has shown MGEs encoding Acr proteins in bacteria like *K. pneumoniae* are 15 times more abundant than MGE free bacteria; and antimicrobial resistance genes (*β*-lactamases and aminoglycoside-modifying enzymes) are overpresented 5 times in MGEs compared to MGE free bacteria, which shows the importance of the relation between Acr proteins and antibiotic resistance genes [37]. A novel Acr protein, AcrEI10, reported in 2024 belongs to a new family of Acr proteins identified from a conjugative plasmid isolated from a CRISPR-Cas positive, MDR *K. pneumoniae* clinical isolate. AcrEI10 is a broad-spectrum Acr protein widely distributed in conjugative plasmids of *Enterobacteriaceae.* It shows an inhibitory effect against CRISPR-Cas system type I-E/I-E* in *K. pneumoniae* and other bacteria and has been proposed to be responsible for spreading antibiotic resistance genes between bacteria. It is encoded mostly by plasmids (most of them contain conjugative modules and antibiotic resistance genes) and with lower frequency in prophages and other regions in bacterial genomes [8]. In the present study, CRISPR-Cas systems were detected in seven of 100 isolates (7%), all of which were MDR, and *cas1* was detected in 13 of 100 isolates (13%).

According to our results, there was no strong inverse or positive correlation between the presence of antibiotic resistance genes and CRISPR-Cas system. Also, *acrEI10* was amplified from one of 100 *K. pneumoniae* isolates (1%), isolate Kp166, which was a CRISPR-Cas positive with antibiotic resistance genes. Analysis of the CRISPR array in this isolate revealed similarities between the spacers and plasmids and *K. pneumoniae* chromosome. As mentioned above, this AcrEI10 protein could be the reason for the copresence of CRISPR-Cas system and antibiotic resistance genes in isolate Kp166 and for other isolates, unknown Acr proteins or other mechanisms could be the reason for this copresence of CRISPR-Cas system and antibiotic resistance genes. Moreover, spacers with similarities to *K. pneumoniae* chromosome in this isolate show the importance of the *acrEI10* presence to protect this bacterium from its own immune system.

Although the impact of CRISPR-Cas on gene transfer between bacteria has been proved to be very small [38], some studies have shown the inverse correlation between the presence of antibiotic resistance genes and CRISPR-Cas system while others are showing no correlation. In a study conducted on 100 *K. pneumoniae* nosocomial isolates in 2023, 32% of the isolates had CRISPR-Cas system and extensively drug resistance rate was higher in CRISPR-Cas negative strains, although the distribution of pandrug resistant strains wasequal both in CRISPR-Cas positive and negative strains [26]. In another survey on 176 clinical isolates of *K. pneumoniae*, 31% of isolates had a CRISPR-Cas system and authors have shown that susceptibility of the isolates toward antibiotics was related to the subtype of CRISPR-Cas system. Among the nine antibiotics tested, isolates positive for the subtype I-E^*^ CRISPR-Cas system were more susceptible to four antibiotics compared with CRISPR-Cas negative isolates, although there was no significant difference between the presence of type I-E CRISPR-Cas and antibiotic susceptibility [39]. According to another research on the association between CRISPR-Cas system and antibiotic resistance in clinical isolates of *K. pneumoniae*, CRISPR positive isolates were more prevalent in MDR isolates in comparison to sensitive isolates. Also, the authors showed there was no significant difference between the presence or absence of CRISPR-Cas system in antibiotic resistant isolates [13]. In another study conducted in Iran, a positive correlation between the presence of *cas1* and some aminoglycoside-resistant genes was detected, although the correlation between *cas3* and some extended-spectrum *β*-lactamases reported to be inverse in clinical isolates of *K. pneumoniae* [11]. In a study in Egypt, it was shown that there was no significant difference between the presence of CRISPR-Cas system in antibiotic susceptible or resistant isolates of *K. pneumoniae*. Moreover, there was no significant correlation between the presence of CRISPR-Cas system and antibiotic resistance genes [15]. According to the present study, no strong correlation between the CRISPR-Cas system and antibiotic resistance genes was observed. However, a weak, positive, and significant correlation was detected between the CRISPR-Cas system and antibiotic resistance genes *bla*_NDM_ and *aac(*6*´)-Ia*. As previously mentioned, Acr proteins and other unknown factors complicate this type of correlation in bacteria. Both *bla*_NDM_ and *aac(*6*´)-Ia* can be carried by plasmids [40,41], and it is possible that plasmids carrying these genes also harbor Acr genes. This co-occurrence could explain the observed weak positive correlation between the CRISPR-Cas system and these specific genes. For instance, isolate Kp166 in this study contained these antibiotic-resistance genes, the CRISPR-Cas system, and *acrEI10*.

This study also revealed a relatively lower prevalence of CRISPR-Cas systems (seven out of 100 isolates, 7%) than reported in some other studies, such as those mentioned above. At first glance, this lower prevalence might seem paradoxical given some literature suggesting an inverse correlation between CRISPR-Cas systems and antibiotic-resistance genes. Firstly, in MDR backgrounds subjected to intense antibiotic selection pressure, the selective advantage may shift towards bacteria harboring MGEs that encode not only antibiotic resistance genes, but also Acr proteins. Secondly, it is conceivable that in MDR *K. pneumoniae*, a fully functional CRISPR-Cas system could be counter-selective by inadvertently targeting and eliminating MGEs that carry essential antibiotic resistance genes required for survival under antibiotic pressure. Furthermore, geographic variation and local adaptation of bacterial populations likely play a significant role, with the specific ecological and evolutionary pressures in the studied referral hospital, potentially favoring a lower intrinsic CRISPR-Cas prevalence in MDR *K. pneumoniae*. While methodological differences including sample sizes across studies cannot be entirely excluded, the multifaceted interplay between Acr proteins, selective pressures in MDR environments, and geographic adaptation likely provide a more compelling explanation for the observed lower CRISPR-Cas prevalence in MDR isolates in this study.

### Conclusions

The present study provides insights into the complex interplay between CRISPR-Cas systems, Acr proteins, and antibiotic resistance in clinical isolates of *K. pneumoniae*. The high prevalence of MDR strains, coupled with the detection of various resistance genes, shows the urgent need for effective strategies to combat these infections. While CRISPR-Cas systems offer a potential defense mechanism against invading MGEs, the emergence of Acr proteins can compromise their effectiveness. Understanding the factors that influence the balance between these systems is crucial for developing novel therapeutic approaches. Future research should prioritize a larger and more diverse sample size, including non-resistant, MDR, XDR, and PDR isolates, to comprehensively understand the mechanisms driving the co-existence of CRISPR-Cas systems and antibiotic resistance genes. Investigating the specific roles of different families of Acr proteins in facilitating the dissemination of resistance will be crucial. Furthermore, it is essential to evaluate the impact of high antibiotic selective pressure environments, such as ICUs, where antibiotic resistance is particularly prevalent, on the activity, evolution, and overall efficacy of CRISPR-Cas systems.

## Supporting information

S1 Table Annealing temperatures and extension times for PCR reactions to amplify targets *khe*, antibiotic resistant genes, CRISPR arrays, *cas1*, *3*, and *acrIE10.* (DOCX)

S1 Data Demographic and source data for each isolate. (XLSX)
