# Supplementary material for: Investigating the impact of type I-E CRISPR-Cas systems and acrEI10 on multidrug-resistance in clinical isolates of Klebsiella pneumoniae
Source: PLoS One. 2025 Nov 19;20(11):e0335756. doi: 10.1371/journal.pone.0335756 (PMC12629477; doi:10.1371/journal.pone.0335756)
Supplement: S1 Table — (DOCX) [file pone.0335756.s001.docx]

| Target | Annealing temperature (ºC) | Extension time  (s) | Amplicon size  (bp) |
| --- | --- | --- | --- |
| *khe* | 58 | 30 | 250 |
| *bla*_VIM_ | 56 | 30 | 865 |
| *bla*_NDM_ | 58 | 45 | 621 |
| *bla*_KPC_ | 60 | 40 | 798 |
| *bla*_OXA-48_ | 58 | 45 | 438 |
| *aph(3´)-Ia* | 57 | 35 | 624 |
| *aac(6´)-Ia* | 61 | 30 | 482 |
| *ant(2″)-Ia* | 67 | 45 | 572 |
| *aac(3)-IIa* | 66 | 35 | 563 |
| *ant(4´)-IIa* | 68 | 45 | 423 |
| *aac(3)-Iva* | 58 | 30 | 314 |
| *aac(3)-Ia* | 61 | 30 | 464 |
| *cas1* | 55 | 30 | 208 |
| *cas3* | 55 | 60 | 620 |
| I-E CRISPR1 | 60 | 70 | Variable |
| I-E* CRISPR2 | 60 | 70 | Variable |
| I-E* CRISPR3 | 60 | 70 | Variable |
| *acrIE10* | 57 | 30 | 212 |
